# Retrospective evaluation of dosimetric quality for prostate carcinomas treated with 3D conformal, intensity modulated and volumetric modulated arc radiotherapy

**DOI:** 10.1002/jmrs.24

**Published:** 2013-11-19

**Authors:** Scott B Crowe, Tanya Kairn, Nigel Middlebrook, Brendan Hill, David R H Christie, Richard T Knight, John Kenny, Christian M Langton, Jamie V Trapp

**Affiliations:** 1 Science and Engineering Faculty, Queensland University of TechnologyBrisbane, Queensland, Australia; 2 Premion, Wesley Medical CentreBrisbane, Queensland, Australia; 3 Australian Clinical Dosimetry Services, Australian Radiation Protection and Nuclear Safety AgencyMelbourne, Victoria, Australia

**Keywords:** Dosimetric quality, intensity modulated radiotherapy, prostate carcinoma, volumetric modulated arc therapy

## Abstract

**Introduction:**

This study examines and compares the dosimetric quality of radiotherapy treatment plans for prostate carcinoma across a cohort of 163 patients treated across five centres: 83 treated with three-dimensional conformal radiotherapy (3DCRT), 33 treated with intensity modulated radiotherapy (IMRT) and 47 treated with volumetric modulated arc therapy (VMAT).

**Methods:**

Treatment plan quality was evaluated in terms of target dose homogeneity and organs at risk (OAR), through the use of a set of dose metrics. These included the mean, maximum and minimum doses; the homogeneity and conformity indices for the target volumes; and a selection of dose coverage values that were relevant to each OAR. Statistical significance was evaluated using two-tailed Welch's *T*-tests. The Monte Carlo DICOM ToolKit software was adapted to permit the evaluation of dose metrics from DICOM data exported from a commercial radiotherapy treatment planning system.

**Results:**

The 3DCRT treatment plans offered greater planning target volume dose homogeneity than the other two treatment modalities. The IMRT and VMAT plans offered greater dose reduction in the OAR: with increased compliance with recommended OAR dose constraints, compared to conventional 3DCRT treatments. When compared to each other, IMRT and VMAT did not provide significantly different treatment plan quality for like-sized tumour volumes.

**Conclusions:**

This study indicates that IMRT and VMAT have provided similar dosimetric quality, which is superior to the dosimetric quality achieved with 3DCRT.

## Introduction

Because the prostate is surrounded by radiosensitive organs and load-bearing bones, there is growing interest in delivering radiotherapy treatments to prostate carcinomas using inverse-planned intensity modulated radiotherapy (IMRT)^1^ and volumetric modulated arc therapy (VMAT),^2^ which are expected to provide improved organ at risk (OAR) dose sparing compared with conventional three-dimensional conformal radiotherapy (3DCRT) treatments. Given that there are differences in treatment planning and delivery efficiency between IMRT, VMAT and 3DCRT,^2^–^4^ it is important to examine the dosimetric quality achieved with intensity modulated techniques and review any differences observed between the modalities.

Reports of the improved OAR sparing achievable with IMRT have often been based on prospective studies, where IMRT and 3DCRT treatments are planned for small numbers of patients and the resulting dose distributions are compared.^5^–^9^ A study by Zelefsky et al.^5^ reported that IMRT allowed reduced rectal and bladder doses and improved coverage compared to 3DCRT in a study of 20 concomitantly planned treatments. Luxton et al.^6^ found that accurately delivered IMRT for prostate cancer can limit dose to normal tissue. Hardcastle et al.^9^ found that IMRT allowed rectal dose reductions.

Similarly, reports of the improved dose distributions achievable with VMAT have largely relied upon prospective comparisons against IMRT using a small number of treatment plans and have produced some contradictory results.^4^,^10^–^13^ For example, Yoo et al.^4^ found that conventional IMRT performed better than RapidArc (the VMAT implementation of Varian Medical Systems, Palo Alto, CA) in bladder, rectum and small bowel dose sparing while obtaining comparable coverage of the planning target volume (PTV) in a study using data from 10 patients. By contrast, Kjær-Kristofferson et al.^10^ found that the RapidArc optimization algorithm provided better or equal sparing of OAR compared to IMRT plans, with decreased target dose homogeneity, in a study of eight prostate cancer patients. Jacob et al.^11^ found RapidArc achieved greater rectum and bladder dose sparing while achieving similar PTV dose homogeneity as dynamic IMRT and helical tomotherapy in a study of nine patients. Similarly, Hardcastle et al.*'*s^12^ 10-patient study found that a VMAT technique provided improved rectal dose sparing compared to standard IMRT. Sze et al.^13^ found that RapidArc could provide greater dosimetric quality than IMRT in a study of 14 patients.

Two studies have compared 3DCRT with IMRT and VMAT. Wolff et al.^8^ found that IMRT, VMAT and serial tomotherapy offered improved quality plans for a set of nine patients. A study by Palma et al.^7^ compared achievable dose distributions for 3DCRT, IMRT and VMAT techniques over 10-patient computed tomography (CT) data sets, finding that IMRT and VMAT treatments offered greater dose reduction in critical organs, and that a variable dose rate VMAT technique provided the greatest dosimetric quality.

A small number of retrospective examinations of delivered VMAT plans have been reported.^3^,^14^ Pesce et al.^14^ examined treatment plans for 45 prostate cases and found that VMAT prostate treatments were meeting improved clinical objectives, but did not compare calculated dose metrics with those of other treatment modalities. Aznar et al.^3^ found that VMAT resulted in a significantly lower dose to the rectum at the expense of PTV coverage in a mean DVH comparison between 46 VMAT treatment plans and 50 independent IMRT plans.

While there are many prospective studies in the literature about the quality of treatment plans, retrospective studies further validate the predictions derived from these prospective analyses.

Retrospective analyses of treatments delivered to patients provide valuable examples of the clinical application of different treatment modalities.

This study retrospectively examines a large number of prostate treatments planned and delivered using 3DCRT, IMRT and VMAT, in terms of an extended list of dose metrics, in order to provide a detailed and robust illustration of the plan quality that may be achieved clinically, using these modalities. The number of patients involved is greater, by an order of magnitude than any cohort examined in previously published prospective or retrospective plan quality studies, and the set of dose metrics evaluated is more comprehensive than any previously applied. This analysis allows objective assessment of the quality of the plans and thereby provides an indication of the advantages and limitations of the 3DCRT, IMRT and VMAT radiotherapy techniques as used at the centres involved in this study.

## Methods

### Patient plans

The treatments were planned on the Eclipse treatment planning system (Varian Medical Systems) version 8.6, using the anisotropic analytical algorithm (AAA) dose calculation engine. The patient group received prostate cancer treatments contemporaneously planned and delivered over a 2-year period (January 2010 through February 2012), on seven dosimetrically matched linear accelerators located at five centres operated by Premion in Queensland, Australia. The Queensland University of Technology (QUT) University Human Research Ethics Committee (HREC) assessed this research as meeting the conditions for exemption from HREC review and approval in accordance with section 5.1.22 of the National Statement on Ethical Conduct in Human Research (2007).^15^

A total of 163 treatment plans were selected for analysis: 83 treated with 3DCRT, 33 treated with IMRT and 47 treated with VMAT (specifically RapidArc). A further 71 plans were excluded due to deviations from the standard planning process. Plans were excluded when the treatment intent was not curative or radical; brachytherapy had been used in conjunction with external beam therapy; altered beam arrangements had been used, for example, where the treatment was altered due to machine servicing or failures; and where there were significant artefacts in the CT data due to hip prostheses. Treatments of prostate beds were included.

Variations in Gleason score and clinical target volume (CTV, measured in cc) were evaluated using a series of two-tailed Welch's *T*-tests to establish whether there was significant variation between the three treatment modality cohorts.

The 3DCRT treatments typically contained six beams: a lateral opposed pair (90° and 270°), an anterior oblique pair (30° and 330°) and a posterior oblique pair (125°–135° and 225–235°), frequently with wedges to compensate for the patient anatomy. The most common prescription was 70 Gy to the intact prostate (45% of patients). Alternative prescriptions for treatments of the prostate bed following a radical prostatectomy were 64 Gy for adjuvant treatments (38% of patients) and 66 Gy for salvage treatments (15% of patients).

The IMRT treatments generally contained either five or seven beams: an anterior beam (0°), an anterior oblique pair (45° and 315°) and a posterior oblique pair (100° and 260°). The PTV dose prescriptions were 78 Gy for intact prostates and 66 Gy (1 patient) for treatments of the prostate bed.

The majority of the VMAT treatments utilized two 360° arcs, rotating in opposite directions. The prescription for intact prostates was generally 78 Gy, with prescriptions of 66 Gy (1 patient), 70 Gy (3 patients) and 74 Gy (15 patients) delivered to prostate beds.

Prescriptions were defined in terms of the dose to an ICRU (International Commission on Radiation Units & Measurements) prescription point,^16^ with a minimum coverage dose of 95% and a maximum accepted hotspot dose of 107%. Where a volumetric prescription was specified the prescription was scaled accordingly. The prescription doses were therefore approximately equal to the PTV median dose values.

The PTVs were defined using a 10-mm expansion to the CTVs, without scalloping around the rectum. The rectal volume was contoured from the inferior end of the sigmoid colon, down to the superior end of the anus, at the pelvic floor. For inverse planning (i.e., for IMRT and VMAT) the rectal volume minus the PTV volume was used for dose optimization. Data presented here are for the whole-rectal volume. Patients were imaged with full bladders and empty rectums.

All treatment plans examined in this study were contemporaneously devised by trained planning staff who were unaware that their work would be used in any subsequent retrospective analysis. Each modality was utilized over the whole 2-year period. Patient selection for each modality was determined by the availability of the different modalities at each centre. (VMAT was available at two centres and IMRT at another two centres.)

### Dose metrics

Treatment plan data were exported from the Eclipse treatment planning system using the DICOM export functionality, producing an RTPLAN file (containing beam parameters), an RTSTRUCT file (containing volume definitions) and an RTDOSE file (containing the calculated dose distribution). The dose assessment metrics were extracted with TADA (Treatment and Dose Assessor), an expansion to the in-house MCDTK (Monte Carlo DICOM ToolKit) software suite.^17^,^18^

The TADA software utilizes the mDCM DICOM library to parse the information stored in the DICOM files. The calculated dose values in each of the volumes of interest were selected by scanning through the RTDOSE distribution, recording values contained in the structure. Determining whether a voxel dose value should be included in the volume population was done using a ray-casting point-in-polygon algorithm,^19^ where the polygons were defined as the contours resulting from CT-slice segmentation (as stored in the RTSTRUCT files).

The TADA processing of the DICOM data produced text files for each patient, presenting the dose metrics for each volume defined for the patient CT data set, the dose metrics for that volume, the conformity indices for the plan and a report on whether dose objectives had been met.

The dose metrics exported in these files were selected on a per-structure basis: for treatment volumes the minimum, near-minimum (*D*_98%_), median, near-maximum (*D*_2%_) and maximum doses were included; for OAR volumes, clinically relevant metrics were also included.

Two OAR guidelines were adopted from objectives presented by Marks et al.^20^ and Michalski et al.^21^ as part of the QUANTEC analysis (Quantitative Analyses of Normal Tissue Effects in the Clinic) of normal tissue effects: that no more than 35% of the rectum should receive 60 Gy (*V*_60Gy_ ≤ 35%) and no more than 20% of the rectum should receive 70 Gy (*V*_70Gy_ ≤ 20%). The 40 Gy rectal coverage was also evaluated, to provide an additional comparison point, which corresponded to Hansen and Roach's^22^ sample dose constraint, that no more than 50% of the rectum should receive 40 Gy (*V*_40Gy_ ≤ 50%). Dose to the femoral heads was evaluated in terms of the recommendation in Lawton et al.*'*s^23^ Radiation Therapy Oncology Group (RTOG) prostate radiotherapy trial consensus report that no more than 5% of the femoral heads should receive 50 Gy (*V*_50Gy_ ≤ 5%). For each treatment modality, the percentage of plans meeting these recommendations was evaluated, as was the mean coverage dose at each of the relevant dose levels.

Planning target volume dose values were averaged over all patients treated with each treatment modality, after being normalized against the patient's ICRU prescription point dose. Individual OAR dose values were normalized against the patient's median PTV doses before averaging. These normalizations allowed the OAR dose sparing provided by the treatments to be compared despite the differences in the PTV prescriptions. A comparison without this normalization of OAR dose was performed for IMRT and VMAT treatments with 78 Gy PTV coverage prescriptions.

A homogeneity index (HI) was used to evaluate the heterogeneity of dose to the treatment volumes, which was defined as^16^:


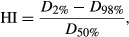


where a result approaching zero indicates that the absorbed dose distribution is near homogeneous.

The equivalent uniform doses (EUDs)^24^,^25^ in the prostate and femoral heads were calculated as:


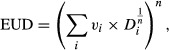


where *v*_*i*_ is the partial volume receiving dose *D*_*i*_, and *n* describes the volumetric dependence of the dose–response relationship. The *n* values used in this study were taken from Burman et al.^26^: 0.12 for the rectum and 0.25 for the femoral heads.

Two different conformity indices were calculated: the RTOG conformity index (CI),^27^ a ratio of the volume of the reference isodose and the target volume; and the van't Riet conformation number (CN),^28^ which takes into account both target volume and healthy tissue irradiation.^29^ The CN was defined as:


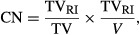


where TV is the target volume, TV_RI_ is the target volume covered by the reference isodose and *V*_RI_ is the volume of the reference isodose. For both CI and CN a value approaching 1 indicates high conformity.

Mean HIs, EUDs, CIs and CNs were calculated for each group of patients treated with each radiotherapy modality. The large data set used in this study allows calculated metrics to be stratified in terms of target size. Small CTVs were defined as being less than 50 cc, medium being 50–70 cc and large being greater than 70 cc.

Confidence limits were evaluated as the standard deviation from each mean. The significance of the statistical variations between the three groups were expressed as *P*-values calculated using two-tailed Welch's *T*-tests for each modality pair (3DCRT vs. IMRT, 3DCRT vs. VMAT, and IMRT vs. VMAT). Smaller *P*-values suggest that the differences between the data sets are unlikely to have arisen by random fluctuations.

## Results

The results of the dose assessments across the entire cohort are presented in Tables 1 and 2. Table 3 presents results stratified according to CTV size.

**Table 1:** Summary of patient cohort information and dose metrics.

Averaged parameter	3DCRT	IMRT	VMAT	P_3DCRT-IMRT_	P_3DCRT-VMAT_	P_IMRT-VMAT_
Number of patients	83	33	47	–	–	–
Gleason score	7.4 ± 0.8	7.4 ± 0.9	7.7 ± 1.0	0.705	0.049	0.238
Primary grade	3.6 ± 0.5	3.5 ± 0.5	3.6 ± 0.6	0.095	0.745	0.245
Secondary grade	3.6 ± 0.7	3.8 ± 0.8	4.0 ± 0.7	0.050	<0.001	0.428
Clinical target volume (cc)	62 ± 23	52 ± 22	56 ± 20	0.065	0.185	0.487
Prescription dose (Gy)	68 ± 3	78 ± 2	76 ± 3	<0.001	<0.001	0.001
Monitor units (MU)	323 ± 28	976 ± 187	783 ± 144	<0.001	<0.001	<0.001
MU efficiency (MU/Gy)	4.7 ± 0.4	12.6 ± 2.7	10.3 ± 2.0	<0.001	<0.001	<0.001
Treatment volume						
Minimum dose^1^	0.95 ± 0.03	0.90 ± 0.10	0.95 ± 0.02	0.021	0.949	0.020
*D*_98%_ dose^1^	0.97 ± 0.02	0.96 ± 0.05	0.97 ± 0.02	0.150	0.805	0.185
Median dose^1^	1.00 ± 0.01	1.01 ± 0.01	1.00 ± 0.02	0.286	0.867	0.354
*D*_2%_ dose^1^	1.02 ± 0.02	1.04 ± 0.01	1.03 ± 0.02	<0.001	0.025	0.152
Maximum dose^1^	1.03 ± 0.02	1.06 ± 0.02	1.05 ± 0.02	<0.001	<0.001	0.685
Homogeneity index	0.05 ± 0.02	0.08 ± 0.05	0.06 ± 0.01	0.003	<0.001	0.044
Rectum						
Median dose^2^	0.65 ± 0.16	0.32 ± 0.08	0.40 ± 0.12	<0.001	<0.001	0.002
*V*_70Gy_ coverage	(3 ± 7)%	(11 ± 4)%	(11 ± 6)%	<0.001	<0.001	0.950
*V*_60Gy_ coverage	(33 ± 12)%	(16 ± 4)%	(17 ± 8)%	<0.001	<0.001	0.198
*V*_40Gy_ coverage	(55 ± 15)%	(28 ± 4)%	(33 ± 14)%	<0.001	<0.001	0.024
Femoral head						
Median dose^2^	0.58 ± 0.08	0.33 ± 0.09	0.28 ± 0.09	<0.001	<0.001	<0.001
*V*_50Gy_ coverage	(1 ± 4)%	(1 ± 1)%	(0 ± 1)%	0.082	0.005	0.056
Conformity indices						
RTOG conformity	1.67 ± 0.18	1.16 ± 0.14	1.21 ± 0.10	<0.001	<0.001	0.070
van't Riet conformity	0.60 ± 0.06	0.82 ± 0.09	0.82 ± 0.05	<0.001	<0.001	0.838

Values represent mean (±1 SD) over the entire patient cohort for each modality. *P*-value (significance) was calculated using two-tailed Welch's *T*-test. 3DCRT, three-dimensional conformal radiation therapy; IMRT, intensity modulated radiation therapy; VMAT, volumetric modulated arc therapy; *D*_*n*%_, dose received by *n*% of volume; *V*_*n*_, percentage of volume receiving dose *n*; EUD, equivalent uniform dose; RTOG, Radiation Therapy Oncology Group.

1 Normalized against patient prescription dose before averaging.

2 Normalized against patient median PTV dose before averaging.

**Table 2:** Summary of adherence to OAR dose constraints.

Averaged parameter (%)	3DCRT (%)	IMRT (%)	VMAT (%)
Rectum			
*V*_70Gy_ < 20	95	100	91
*V*_60Gy_ < 35	56	100	96
*V*_40Gy_ < 50	27	100	87
Femoral head			
*V*_50Gy_ < 5	90	97	98

Values represent the percentage of plans adhering to the OAR dose coverage constraints. OAR, organs at risk; 3DCRT, three-dimensional conformal radiation therapy; IMRT, intensity modulated radiation therapy; VMAT, volumetric modulated arc therapy; *V*_*n*_, percentage of volume receiving dose *n*.

**Table 3:** Summary of small, medium and large CTV cohort dose metrics.

Averaged parameter	3DCRT	IMRT	VMAT	P_3D-IMRT_	P_3D-VMAT_	P_IMRT-VMAT_
Number of patients						
Small CTV cohort	23	14	13	–	–	–
Medium CTV cohort	16	7	16	–	–	–
Large CTV cohort	24	4	5	–	–	–
Median PTV dose^1^						
Small CTV cohort	0.99 ± 0.01	1.01 ± 0.01	1.01 ± 0.02	0.005	0.029	0.746
Medium CTV cohort	1.00 ± 0.01	1.00 ± 0.02	1.00 ± 0.02	0.765	0.282	0.357
Large CTV cohort	1.01 ± 0.01	1.00 ± 0.01	1.00 ± 0.03	0.127	0.975	0.583
PTV homogeneity index						
Small CTV cohort	0.04 ± 0.01	0.09 ± 0.08	0.07 ± 0.01	<0.001	0.042	0.308
Medium CTV cohort	0.05 ± 0.01	0.08 ± 0.01	0.06 ± 0.01	<0.001	0.002	0.034
Large CTV cohort	0.06 ± 0.04	0.07 ± 0.02	0.06 ± 0.01	0.590	0.733	0.407
Median rectal dose^2^						
Small CTV cohort	0.57 ± 0.17	0.33 ± 0.10	0.40 ± 0.16	<0.001	0.005	0.184
Medium CTV cohort	0.73 ± 0.14	0.31 ± 0.09	0.43 ± 0.10	<0.001	<0.001	0.017
Large CTV cohort	0.73 ± 0.13	0.33 ± 0.07	0.45 ± 0.09	<0.001	<0.001	0.057
Median femoral dose^2^						
Small CTV cohort	0.58 ± 0.06	0.32 ± 0.08	0.26 ± 0.07	<0.001	<0.001	0.006
Medium CTV cohort	0.57 ± 0.07	0.28 ± 0.14	0.31 ± 0.06	<0.001	<0.001	0.817
Large CTV cohort	0.60 ± 0.11	0.38 ± 0.03	0.34 ± 0.05	<0.001	<0.001	0.075
RTOG conformity						
Small CTV cohort	1.59 ± 0.13	1.17 ± 0.11	1.24 ± 0.12	<0.001	<0.001	0.122
Medium CTV cohort	1.69 ± 0.20	1.08 ± 0.22	1.18 ± 0.08	<0.001	<0.001	0.306
Large CTV cohort	1.72 ± 0.19	1.11 ± 0.04	1.19 ± 0.13	<0.001	<0.001	0.250
van't Riet conformity						
Small CTV cohort	0.63 ± 0.05	0.81 ± 0.11	0.80 ± 0.07	<0.001	<0.001	0.854
Medium CTV cohort	0.60 ± 0.06	0.82 ± 0.09	0.83 ± 0.04	<0.001	<0.001	0.720
Large CTV cohort	0.58 ± 0.07	0.90 ± 0.03	0.83 ± 0.07	<0.001	<0.001	0.082

Values represent mean (±1 SD) over each CTV size (small <50 cc, medium 50–70 cc, and large >70 cc) cohort for each modality. *P*-value (significance) was calculated using two-tailed Welch's *T*-test. 3DCRT, three-dimensional conformal radiation therapy; IMRT, intensity modulated radiation therapy; VMAT, volumetric modulated arc therapy; CTV, clinical target volume; PTV, planning target volume; RTOG, radiation therapy oncology group.

1 Normalized against patient prescription dose before averaging.

2 Normalized against patient median PTV dose before averaging.

There was no significant difference between the Gleason scores and CTVs for the 3DCRT, IMRT and VMAT cohorts (*P* > 0.05 in most cases). These results suggest that there was no significant difference between the pathologies treated using the three radiotherapy modalities. The higher mean CTV seen with 3DCRT is due to a difference in the proportion of prostate bed treatments.

There was a significant difference between the prescription doses and the number of monitor units (*P* < 0.001) required to deliver these doses across the three modalities. The differences in monitor unit efficiency (the number of MU required to delivery 1 Gy of prescription dose) were statistically significant (*P* < 0.001). The VMAT treatments, on average, required the delivery of fewer monitor units, requiring less treatment delivery time, than IMRT, but both modalities required significantly more monitor units than 3DCRT.

Data in Tables 1 and 3 show that the median PTV and prescription dose ratios for the 3DCRT, IMRT and VMAT treatments were near unity and not significantly different. The differences in the other PTV dose metrics were significant: the IMRT and VMAT treatments showed higher maximum and near-maximum doses (*P* < 0.05 in all cases), and generally greater heterogeneity (*P* < 0.001 and 0.002 for medium CTVs, respectively).

Data in Tables 1 and 2 also indicate that the dose sparing of the rectum and femoral heads was consistently higher for IMRT and VMAT than for the 3DCRT treatments. The data in Table 3 suggest that the rectal dose sparing achievable with 3DCRT decreases with increasing CTV size, a trend not observed for the IMRT and VMAT modalities. The lowest observed femoral head doses were achieved using VMAT and the lowest rectal doses were achieved using IMRT. *T*-test results shown in Table 3 indicate that the differences between these OAR doses are significant when 3DCRT is compared to either IMRT or VMAT (*P* < 0.001 for rectums and femoral heads in most cases).

The results of the dose assessments for IMRT and VMAT treatments involving the delivery of a 78 Gy PTV coverage prescription are presented in Table 4. A statistically significant difference in femoral head dose sparing (*P* < 0.001 for median dose) can be observed. Table 3 suggests that any differences between IMRT and VMAT dose sparing, in terms of both rectal and femoral median dose values, are not statistically significant when like-sized CTVs are compared. Table 3 also suggests that there is no significant difference in conformity between IMRT and VMAT.

**Table 4:** Summary of 78 Gy prescription cohort dose metrics.

Averaged parameter	IMRT	VMAT	*P*
Number of patients	31	27	–
Clinical target volume (cc)	52 ± 22	51 ± 19	0.690
Monitor units (MU)	967 ± 172	768 ± 117	<0.001
MU efficiency (MU/Gy)	12.4 ± 2.2	9.9 ± 1.5	<0.001
Treatment volume			
Minimum dose (Gy)	71.7 ± 3.5	73.9 ± 0.7	0.002
*D*_98%_ dose (Gy)	75.1 ± 1.3	75.0 ± 1.2	0.662
Median dose (Gy)	78.4 ± 0.8	77.6 ± 1.1	0.004
*D*_2%_ dose (Gy)	80.8 ± 1.1	79.7 ± 1.3	0.003
Maximum dose (Gy)	82.4 ± 1.4	81.4 ± 1.3	0.005
Homogeneity index	0.07 ± 0.02	0.06 ± 0.01	0.004
Rectum			
Median dose (Gy)	24.9 ± 6.3	26.6 ± 7.9	0.390
EUD (Gy)	59.0 ± 2.6	59.0 ± 3.5	0.956
*V*_70Gy_ coverage	(11 ± 3)%	(11 ± 5)%	0.995
*V*_60Gy_ coverage	(16 ± 4)%	(16 ± 6)%	0.709
*V*_40Gy_ coverage	(28 ± 4)%	(30 ± 11)%	0.351
Femoral head			
Median dose (Gy)	26.2 ± 7.1	20.9 ± 7.1	<0.001
EUD (Gy)	30.7 ± 3.6	24.5 ± 6.8	<0.001
*V*_50Gy_ coverage	(1 ± 1)%	(0 ± 1)%	0.136
Conformity indices			
RTOG conformity	1.16 ± 0.15	1.21 ± 0.08	0.115
van't Riet conformity	0.83 ± 0.07	0.82 ± 0.04	0.418

Values represent mean (±1 SD) over 78 Gy prescription cohorts for each modality. *P*-values (significance) calculated using two-tailed Welch's *T*-test. IMRT, intensity modulated radiation therapy; VMAT, volumetric modulated arc therapy; *D*%, dose to percentage of volume; *V*_*n*_, percentage of volume with dose; EUD, equivalent uniform dose; RTOG, radiation therapy oncology group.

The conformity scores for IMRT and VMAT listed in Table 1 are similar, with both modalities showing greater conformity than the 3DCRT treatments. Table 3 shows significant differences between 3DCRT and both IMRT and VMAT (*P* < 0.001 for CN) when conformity indices and CNs are evaluated. These results confirm that the high doses in the treatment plans are generally better matched to the target volumes, and provide better avoidance of healthy tissues, in the IMRT and VMAT treatment plans than in the 3DCRT plans.

## Discussion

The results of a retrospective dose assessment of a large number of treated radiotherapy plans (presented in Table 1), stratified for CTV size and PTV prescription (presented in Tables 3 and 4, respectively), support observations made in smaller, prospective and retrospective treatment plan quality studies. The observation that the intensity modulated treatments provide improved OAR sparing with only slightly reduced PTV dose homogeneity compared with conventional 3DCRT treatments, for the 163 cases examined here, confirms observations made in previous 9-, 10- and 20-patient planning studies.^5^,^7^,^8^

The observation of improved OAR sparing with intensity modulated treatments complements Zelefsky et al.'s^5^ extensive comparison of the treatment outcomes achieved using IMRT with 3DCRT. Zelefsky et al.^5^ found that, in a large cohort of 3DCRT and IMRT patients, the risk of several rectal complications was significantly lower in the IMRT patients.

The differences between OAR doses for the 3DCRT and intensity modulated plans examined in this study were sufficiently large that despite a 10% escalation in the mean prescribed dose to the PTV, compared to the 3DCRT treatments, the mean rectal volumes covered by doses of 40 and 60 Gy were approximately halved in the intensity modulated treatments (see Table 1). The 3DCRT treatments, on average, failed to meet Hansen and Roach's^22^ recommendation that less than 50% of the rectum should receive a dose of 40 Gy. Almost all the IMRT and VMAT treatments were able to meet this criterion.

Based on the results shown in Tables 4, neither of the two intensity modulated treatment modalities appears preferable to the other. Smaller studies have shown that preferable OAR sparing can be provided by IMRT plans^4^ or by VMAT plans^9^–^11^ or by each of the two modalities for different OAR.^14^ Results shown in Tables 1 and 4 indicate that while the VMAT treatments appear to provide reduced rectal sparing and improved femoral sparing when all patient plans are examined together, the statistical significance of this result decreases when the patient plans are stratified by target size. This result provides confirmation of the value of delivering intensity modulated treatments using VMAT at the centres involved in this study. For our patient treatments, VMAT provided dose distributions that were similar to IMRT (see Table 3) while using fewer monitor units (see Table 1) and requiring less beam-on time than IMRT. These results also provide confirmation of the value of performing detailed, stratified analyses on large patient data sets.

The evaluation of 163 patient plans should be regarded as one of the strengths of our study design. The use of this large number of patient plans made it possible for data analysis to be stratified in terms of both target volume and prescription dose, to provide more detailed analysis of apparent trends in plan quality than had previously been available. Clearly, the investigation of such a large patient cohort made prospective replanning of the treatments, using modalities other than those with which they were treated, clinically unachievable. Such prospective replanning is an obvious direction for future study. However, we expect that retrospective treatment plan auditing and quality assurance will be the major focus of future studies. It is likely that, as computational tools such as TADA^18^ become more available, automated retrospective analysis of ever larger numbers of treatment plans will become part of routine clinical practice.

## Conclusion

This retrospective dosimetric analysis of 163 clinically delivered 3DCRT, IMRT and VMAT radiotherapy treatment plans, delivered across five centres operated by one organization, has demonstrated that although the PTV dose homogeneity achieved by the IMRT and VMAT plans was slightly reduced, these intensity modulated radiotherapy techniques permitted an improvement in conformity and OAR sparing that makes them preferable for future treatments of prostate carcinomas. For the patient treatment cohort examined in this work, no statistically significant differences in dosimetric quality were observed between IMRT and VMAT across patients with like-sized CTVs. The value of using intensity modulated treatment modalities, such as IMRT and VMAT, is most apparent in their increased compliance with recommended OAR dose constraints, compared to conventional 3DCRT treatments, potentially leading to improved treatment outcomes.
